# Supplementary material for: Bayesian refinement of protein structures and ensembles against SAXS data using molecular dynamics
Source: PLoS Comput Biol. 2017 Oct 18;13(10):e1005800. doi: 10.1371/journal.pcbi.1005800 (PMC5662244; doi:10.1371/journal.pcbi.1005800)
Supplement: S1 Archive — (BZ2) [file pcbi.1005800.s011.tar.bz2 › gromacs_4.62_bayesian_saxs_refi/share/html/online/edi.html]

edi file format


## edi file format

|  |  |
| --- | --- |
| Main Table of Contents | **VERSION 4.6** |
| GROMACS homepage | **Sat 19 Jan 2013** |

---

### Description

Files with the edi file extension contain information for 
mdrun to run Molecular Dynamics with Essential Dynamics constraints.
These files can be generated by the program WEDSAM which uses
output from the programs in the ESSDYN menu of the
WHAT IF program.
  


---

http://www.gromacs.org
